# Modulating M1/M2 macrophage polarization with plant metabolites: a therapeutic strategy for rheumatoid arthritis

**DOI:** 10.3389/fphar.2025.1651763

**Published:** 2025-10-17

**Authors:** Yuanyuan Yang, Xiaoyu Wang, Youqian Kong, Yang Pan, Xiadong Yang, Ziyi Zhang, Zeguang Li

**Affiliations:** ^1^ Graduate School, Heilongjiang University of Chinese Medicine, Harbin, China; ^2^ Department of Traditional Chinese Medicine, The First Hospital of Lanzhou University, Lanzhou, China; ^3^ First Affiliated Hospital, Heilongjiang University of Chinese Medicine, Harbin, China

**Keywords:** plant metabolite, macrophage polarization, rheumatoid arthritis, mechanism, traditional Chinese medicine

## Abstract

Rheumatoid arthritis (RA) is a systemic autoimmune disorder marked by persistent synovitis and the degradation of joint cartilage and surrounding bone due to inflammation. A variety of immune cells, particularly macrophages, are involved in the initiation and maintenance of inflammation in RA, along with leukocyte adhesion and migration, matrix breakdown, and neovascularization. Environmental and internal stimuli drive macrophages to polarize into two major phenotypes: M1, which exerts strong bactericidal effects and contributes to chronic inflammation and tissue injury, and M2, which inhibits inflammation and facilitates tissue repair. The dysregulation of M1/M2 macrophage polarization is a key contributor to the pathogenesis and disease progression of RA. Plant metabolites are typically characterized by multi-component, multi-target, and multi-pathway actions, and their underlying mechanisms may include regulation of immune function, especially the balance of macrophage polarization. Current evidence indicates that such metabolites may provide certain therapeutic benefits and a relatively manageable safety profile in the management of RA and related disorders. In this review, we summarize the potential mechanisms by which various plant metabolites modulate macrophage function and polarization under inflammatory conditions, providing evidence for their clinical application in RA treatment and offering new insights into precision therapy for RA.

## 1 Introduction

Rheumatoid arthritis (RA) is a systemic autoimmune disease characterized primarily by erosive arthritis, with pathological features of persistent synovitis and destruction of articular cartilage and adjacent bone (Smolen et al., 2016; Smith and Berman, 2022). Joint lesions are the predominant clinical feature of RA, but in the course of disease progression, some patients may develop vasculitis or multi-organ involvement (e.g., heart, lungs, kidneys), resulting in systemic injury and severely affecting patient health and quality of life, and is a highly disabling disease (Gravallese et al., 2024). The worldwide prevalence of RA is estimated at 0.5%–1.0%. Its precise etiology and pathogenesis remain incompletely understood, with genetic predisposition, environmental influences, and immune dysregulation collectively involved in disease initiation and progression (McInnes and Schett, 2011). As pivotal components of the innate immune system, macrophages play a dual regulatory role in maintaining immune homeostasis and modulating inflammatory cascades by secreting diverse cytokines, chemokines, and growth factors (Sheu and Hoffmann, 2022). Macrophages are involved in the initiation and maintenance of inflammation, leukocyte adhesion and migration, matrix degradation, and neovascularization in RA (Zheng et al., 2024). Driven by exogenous pathogens and endogenous cytokine regulation, macrophages predominantly polarize into two distinct functional phenotypes: M1 and M2 (Chambers et al., 2020). M1, which secretes pro-inflammatory cytokines such as TNF-α and IL-6 to promote inflammation and tissue damage; and M2, which releases anti-inflammatory cytokines like IL-10 and TGF-β to suppress inflammation and promote tissue repair (Locati et al., 2020). Recent studies have revealed that the imbalance of M1/M2 phenotypes and the resulting disruption of the immune microenvironment constitute one of the key mechanisms driving the persistent progression of chronic inflammation and bone destruction in RA (Hanlon et al., 2024; Yang et al., 2024). Consequently, modulation of macrophage polarization to re-establish immune homeostasis has become one of the new therapeutic approaches for RA.

At present, the clinical management of RA primarily aims to suppress inflammation, relieve symptoms, slow joint structural destruction, and maintain systemic function. Commonly used medications include nonsteroidal anti-inflammatory drugs (NSAIDs), disease-modifying antirheumatic drugs (DMARDs), biologics, and glucocorticoids (Burmester and Pope, 2017). However, long-term use of these drugs is often associated with adverse effects such as infections, hepatic and renal impairment, and metabolic disorders; in addition, some patients may develop primary or secondary drug resistance, limiting their long-term efficacy (Smolen et al., 2023). In this context, designing innovative therapies that are low in toxicity, act on multiple targets, and integrate both immune regulation and tissue repair has emerged as a major focus of RA research.

In recent years, various plant metabolites have shown great promise in the treatment of RA due to their well-defined pharmacological activities and favorable safety profiles. For example, triptolide suppresses the TLR4/NF-κB signaling cascade to significantly decrease M1 macrophage populations, while promoting the expression of M2-related markers (Zhu et al., 2025). Curcumin, by contrast, modulates macrophage repolarization synergistically through activation of the Nrf2/HO-1 pathway and suppression of the NLRP3 inflammasome (Zhang C. et al., 2024; Li Q. et al., 2025). Such plant metabolites act on multiple nodes of the inflammatory network and, in some cases, display synergy with standard DMARDs, offering the prospect of dose reduction and fewer side effects (Wang X. et al., 2025). In terms of clinical translation, several nanocarrier systems based on plant metabolites (such as liposomes and polymeric micelles) have been developed to enhance targeting efficiency toward macrophages and reduce systemic exposure risks. Some formulations have completed preclinical evaluations, showing promising translational potential (Huang et al., 2024; Deng et al., 2025).

This article focuses on the biological basis of macrophages, the signaling networks of polarization, and their regulatory mechanisms in RA. Incorporating the latest research advances, it systematically elucidates how plant metabolites influence the M1/M2 polarization balance by modulating key signaling pathways and epigenetic modifications, aiming to provide new theoretical foundations and research directions for developing RA therapies based on immune microenvironment regulation.

## 2 The role of macrophage polarization in RA

### 2.1 M1 macrophages

M1 macrophages are differentiated through classical activation pathways involving interferon (IFN)-γ, lipopolysaccharide (LPS), and granulocyte-macrophage colony-stimulating factor (GM-CSF). They secrete pro-inflammatory cytokines such as IL-1, IL-6, IL-12, and TNF-α, along with various chemokines, thereby amplifying local inflammation and promoting Th1/Th17 immune responses (Ma et al., 2019; Orecchioni et al., 2019). They highly express co-stimulatory molecules such as MHC II and CD80/CD86, exhibit strong antigen-presenting capacity, and can effectively activate T cell–mediated adaptive immune responses (Kang and Kumanogoh, 2020). Moreover, M1-type macrophages enhance the expression of suppressor of cytokine signaling 3 (SOCS3) and activate inducible nitric oxide synthase (iNOS), leading to the production of mediators like ROS and RNS. While endowing strong antimicrobial activity, this can also intensify chronic inflammation and tissue injury (Shapouri-Moghaddam et al., 2018).

### 2.2 M2 macrophages

M2 macrophages differentiate via alternative pathways under the induction of anti-inflammatory cytokines such as IL-4, IL-10, and IL-13 (Satoh and Akira, 2013; Wang et al., 2021). They are characterized by high expression of protein markers such as CD10, CD163, and CD206, as well as arginase-1 (Arg-1), chitinase-like protein 3 (Chil3), and the cysteine-rich secretory protein FIZZ (Chen et al., 2019). M2 macrophages suppress inflammation by secreting IL-4, IL-10, and IL-13, promote collagen deposition and tissue remodeling via TGF-β and Arg-1, and facilitate tissue repair and angiogenesis through growth factors and cytokines such as epidermal growth factor (EGF), basic fibroblast growth factor (bFGF), and angiopoietin (Ang), playing a key role in maintaining immune homeostasi (Willenborg et al., 2012; Dey et al., 2014; Zhao et al., 2023).

**FIGURE 1:**
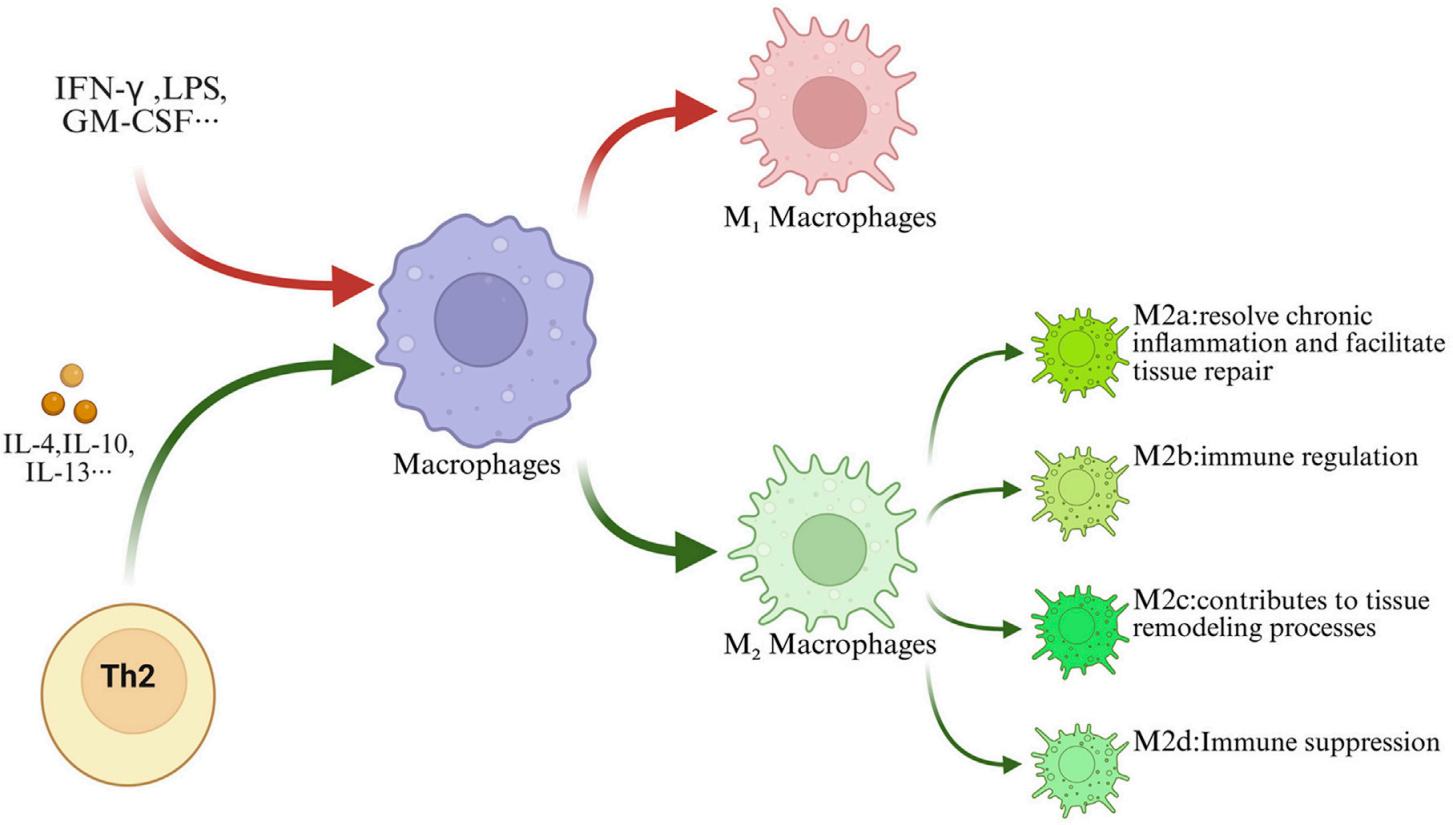
Macrophage polarization. Abbreviations: Under the influence of exogenous pathogen stimulation and endogenous cytokine regulation, macrophages can differentiate mainly into two functionally distinct phenotypes: M1 and M2. And M2 macrophages further differentiate into M2a, M2b, M2c, and M2d subtypes.

Based on their induction mechanisms and functional differences, M2 macrophages can be further classified into four subtypes: M2a (wound-healing type, induced by IL-4/13, secretes anti-inflammatory cytokines such as IL-10 and TGF-β, promoting repair), M2b (regulatory type, induced by immune complexes or TLR agonists, exerting immunoregulatory functions), M2c (deactivated type, induced by IL-10/TGF-β, involved in tissue remodeling), and M2d (tumor-associated type, activated by growth factors, possessing immunosuppressive properties) (Asmis, 2016; Yue et al., 2017; Smigiel and Parks, 2018; Anders et al., 2022; Hung et al., 2024; Cutolo et al., 2025) (Figure 1).

### 2.3 The relationship between M1/M2 macrophage polarization balance and RA

Imbalance between M1 and M2 polarization is a central mechanism driving joint inflammation and bone destruction in RA (Cutolo et al., 2022). Tardito S and colleagues (Tardito et al., 2019) found that in patients with active RA, M1 macrophages are significantly increased in the synovium and peripheral blood, whereas during remission, the proportion of M2 macrophages rises. *In vivo* studies have shown that in the RA synovial microenvironment, numerous cytokines, chemokines, growth factors, and other key molecules form a positive feedback loop that promotes M1 polarization while inhibiting M2 functions, driving chronic inflammation and pannus formation (Tao et al., 2023). Evidence from clinical research indicates that RA patient synovial tissues exhibit elevated expression of M1 markers (CD86, iNOS, TNF-α) and reduced expression of M2 markers (CD163, IL-10), with M1 abundance positively associated with DAS28 scores and severity of bone erosion (Zhou et al., 2024). It is evident that M1 macrophages directly contribute to cartilage destruction by activating synovial fibroblasts and promoting the release of matrix metalloproteinases (MMPs).

**FIGURE 2:**
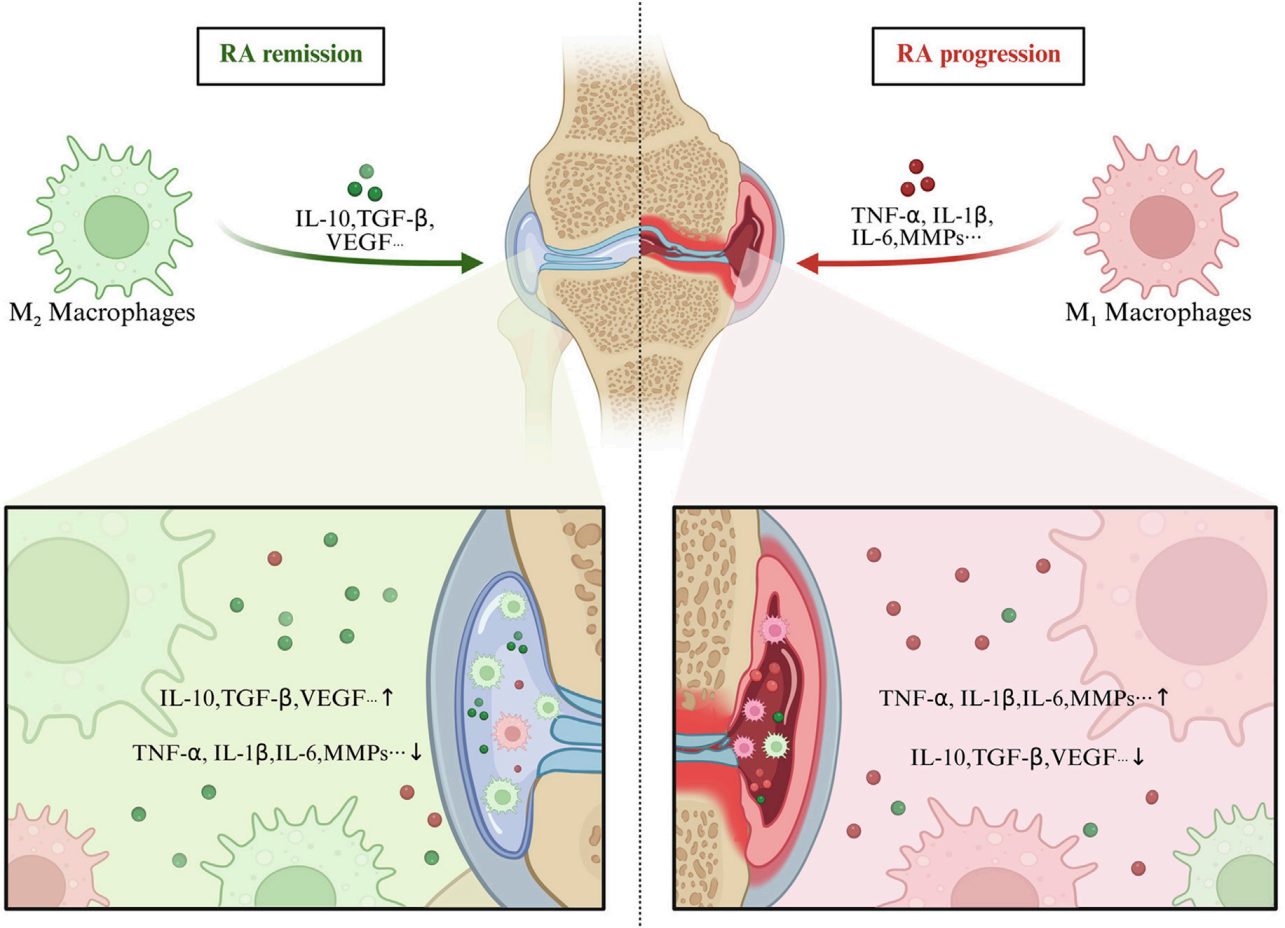
The relationship between M1/M2 macrophage polarization balance and RA. Abbreviations: Overactivation of M1 macrophages results in excessive production of pro-inflammatory cytokines, which exacerbates synovial inflammation and joint bone erosion. Conversely, insufficient M2 polarization fails to suppress inflammation effectively or promote tissue repair.

Lin et al. (2023) demonstrated that the main plant metabolites of the traditional Chinese formula *Wutou Decoction* (aconitine, astragaloside IV, ephedrine, paeoniflorin, and glycyrrhizic acid) can act in combination to modulate the NF-κB/PPARγ pathway, rebalance M1/M2 polarization, and ameliorate synovial inflammation in RA. According to Peng et al. (2019) enhancing M2 macrophage polarization inhibits inflammatory responses and reduces joint injury. JAK inhibitors, such as tofacitinib, reshape macrophage balance by inhibiting M1-associated IFN-γ–STAT1 signaling while preserving M2-related IL-4–STAT6 pathways, leading to significant improvement of RA symptoms (Fleischmann et al., 2023). Therefore, promoting the transition from M1 to M2 phenotypes has emerged as a novel therapeutic strategy for RA, with significant clinical potential (Figure 2).

## 3 Targeting M1/M2 macrophage polarization with plant metabolites in the treatment of RA

**FIGURE 3:**
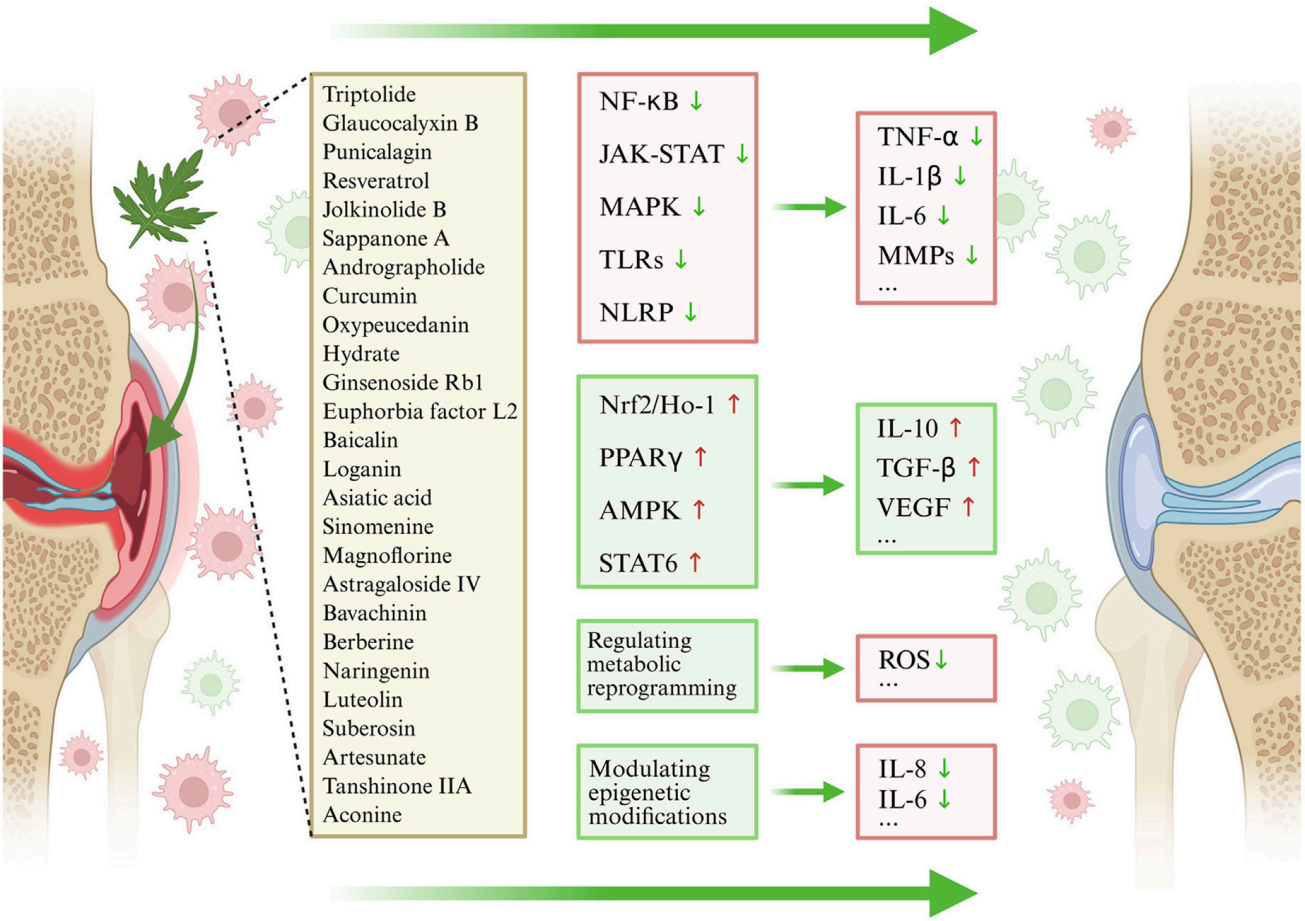
Targeting M1/M2 Macrophage Polarization with plant metabolites in the Treatment of RA. Abbreviations: Plant metabolites maintain the balance between M1 and M2 macrophages by inhibiting core pro-inflammatory pathways to reduce M1 polarization, activating core anti-inflammatory/antioxidant pathways to promote M2 polarization, regulating metabolic reprogramming, and modulating epigenetic modifications, ultimately improving synovial inflammation in RA.

**FIGURE 4:**
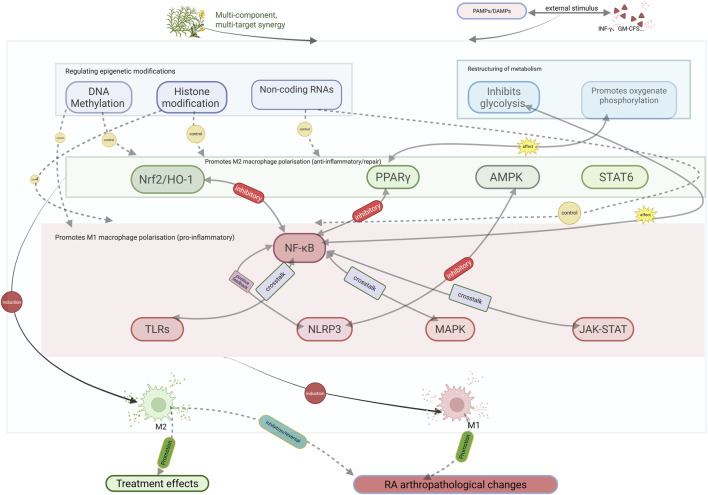
Plant metabolites regulate macrophage polarization to treat RA through multi-target, multi-pathway mechanisms. Abbreviations: Plant metabolites with defined biological activity influence M1 and M2 macrophage polarization by modulating key signaling pathways and epigenetic modifications. Excessive M1 polarization leads to the final pathological changes in RA, whereas M2 polarization can reverse or suppress RA joint pathology and promote its treatment.

In recent years, numerous studies have demonstrated that plant metabolites exhibit considerable therapeutic potential in various diseases by regulating macrophage polarization (Chen et al., 2024). To systematically evaluate the relevant evidence, this study, based on the PRISMA guidelines, reviewed experimental and clinical studies published in recent years in databases such as PubMed, Web of Science, and CNKI concerning plant metabolites (with their botanical origins taxonomically validated via http://www.plantsoftheworldonline.org), macrophage polarization, and RA. The review addresses molecular mechanisms, pharmacodynamics, and clinical translational potential. These mechanisms involve multiple signaling pathways and molecular targets, and the synergistic actions across such multi-pathway, multi-target networks provide a new perspective for precision therapy of RA and other inflammatory diseases. Furthermore, the methodological rigor of the included *in vitro* and *in vivo* studies was evaluated using the Consensus-based Phytochemical Methodology Program (ConPhyMP) tool (available at https://ga-online.org/best-practice/), which provides a checklist of essential standards for reporting natural product pharmacological research. (Figures 3, 4).

### 3.1 Inhibiting key pro-inflammatory pathways to reduce M1 polarization

#### 3.1.1 Nuclear factor-kappa B (NF-κB) signaling pathway

NF-κB is a transcription factor composed of Rel family proteins (such as p65 and p50), which usually binds to IκB and exists in an inactive form in the cytoplasm (Zhang et al., 2017). Upon endogenous stimulation, IκB kinase (IKK) is activated, leading to the phosphorylation and degradation of IκB, which releases NF-κB to translocate into the nucleus and regulate the transcription of downstream genes, including pro-inflammatory cytokines (e.g., TNF-α, IL-1β, IL-6) and adhesion molecules, thereby participating in diverse biological processes such as inflammation, immune responses, cell proliferation, apoptosis, and differentiation (Guo et al., 2024). In RA, activation of this pathway promotes the expression of proinflammatory cytokines such as TNF-α, IL-1β, and IL-6, strongly induces M1 polarization, and exacerbates synovial inflammation and joint destruction (Marques et al., 2025).

Triptolide, an extract derived from the botanical drug *Tripterygium wilfordii Hook. f.*, not only inhibits the binding of NF-κB to DNA but also cooperatively suppresses the MAPK and STAT1 pathways, thereby reducing proinflammatory cytokine secretion, blocking M1 polarization, and exerting anti-inflammatory, antiproliferative, and immunosuppressive effects. Preclinical studies have demonstrated that Triptolide can significantly reduce arthritis scores and attenuate bone erosion in animal models (Yang et al., 2016; Wang X. et al., 2025). Clinical observations further suggest that formulations derived from these metabolites, such as Tripterygium glycoside tablets, may provide symptomatic relief in RA patients. However, their therapeutic efficacy and safety remain to be substantiated by larger, high-quality clinical trials. Particular attention should be given to their potential hepatotoxicity and nephrotoxicity, highlighting the need for rigorous safety monitoring in clinical application (Zhang Y. et al., 2021; Zhou et al., 2025). Glaucocalyxin B (Gla B), a sesquiterpene extracted from *Isodon japonicus var. glaucocalyx*, has been proven in experiments to suppress NF-κB signaling via direct targeting of p65, significantly lowering TNF-α and IL-1β levels without altering IL-10 or TGF-β1 expression. In models of joint inflammation, it exhibits strong protective effects on cartilage (Han et al., 2021). Punicalagin, a bioactive substance isolated from *Punica granatum L.*, has been shown in CIA models to suppress NF-κB pathway receptor activators, mitigate M1 macrophage polarization and pyroptosis, and markedly relieve joint pathological inflammation (Ge et al., 2022). Importantly, despite their promising effects in preclinical studies, challenges including poor oral bioavailability and rapid metabolic clearance limit their clinical application, highlighting the urgent need for formulation optimization, such as nanocarrier-based delivery systems.

#### 3.1.2 Janus kinase-signal transducer and activator of transcription (JAK-STAT) signaling pathway

The JAK-STAT signaling pathway is a major route for cytokine signal transduction, comprising the JAK family (including JAK1, JAK2, TYK2, and JAK3) and the STAT family (including STAT1, STAT3, STAT4, STAT5a/b), and is broadly involved in regulating immune responses, inflammatory reactions, and various biological processes such as cell proliferation, differentiation, and apoptosis (Samra et al., 2025). In RA, aberrant activation of the JAK-STAT pathway leads to elevated levels of pro-inflammatory cytokines such as TNF-α, IL-1β, and IL-6, exacerbating synovial inflammation and bone destruction, and by regulating osteoblast and chondrocyte functions, promotes bone resorption and cartilage degradation, aggravating joint damage (Hu et al., 2022). It has also been reported that phosphorylation of STAT1 and STAT3 promotes M1 macrophage polarization, with IFN-γ inducing the M1 phenotype through the JAK-STAT1 axis (Fortelny et al., 2024). In addition, through involvement in pain transmission and Th17 cell differentiation, it is intimately linked to chronic pain and bone destruction in RA (Simon et al., 2021).

Resveratrol, a naturally occurring polyphenol, exhibits anti-inflammatory effects; studies have demonstrated that it inhibits M1 polarization by downregulating the JAK1-STAT1/STAT3 pathway and deacetylating NF-κB p65, thereby reducing the secretion of proinflammatory mediators including IL-6 and IL-12, ultimately relieving synovitis in animal models (Oliveira et al., 2017; Wang P. et al., 2024). Jolkinolide B (JB), a diterpene metabolites isolated from *Euphorbia pekinensis Rupr.,* in plant, exhibits anticancer, anti-inflammatory, and antituberculosis properties. Research has demonstrated that JB markedly decreases arthritis scores and synovial hyperplasia in CIA rats by inhibiting the JAK2/STAT3 signaling pathway and reducing inflammatory cytokine levels. It efficiently antagonizes M1 polarization, thereby alleviating inflammation and preventing bone erosion in CIA models (Yan et al., 2024). The mechanism involves JB suppressing mRNA expression of inflammatory cytokines in the ankle joints of CIA rats and reducing their levels in LPS-stimulated RAW264.7 macrophages, effectively inhibiting M1 polarization and significantly downregulating JAK2/STAT3 protein expression. Sappanone A (SA), an active plant metabolites of *Tamarindus indica L.*, possesses anti-inflammatory, antioxidant, and bone-protective properties. Research has shown that SA can downregulate the protein phosphorylation levels of JAK2, STAT3, PI3K, AKT, and p65 *in vivo* and *in vitro*, and reverse the increase in protein phosphorylation levels of PI3K/AKT/NF-κB and JAK2/STAT3 pathway-related proteins induced by agonists, thereby reducing the levels of pro-inflammatory cytokines TNF-α, IL-1β, IL-6, and IL-17. While promoting the levels of the anti-inflammatory cytokine IL-10, inhibiting M1 polarization while promoting M2 polarization, thereby significantly reducing the arthritis index in CIA rats, alleviating paw swelling, and improving inflammatory cell infiltration and cartilage degradation (Deng et al., 2024). Nevertheless, these conclusions largely stem from *in vitro* and animal experiments, with the mechanisms of action yet to be fully elucidated. Their clinical translation and real-world efficacy in humans demand further confirmation in rigorous randomized controlled trials. While JAK-STAT inhibitors are already in clinical use, metabolites from plants, with their multi-target characteristics, may provide superior safety. Further attention should be directed toward their potential synergy with current therapies and their long-term safety profile.

#### 3.1.3 Mitogen-activated protein kinase (MAPK) signaling pathway

The MAPK signaling pathway is also one of the key intracellular signal transduction cascades. Studies have shown that aberrant activation of the MAPK pathway can enhance the expression of M1-associated genes (such as iNOS and COX-2), leading to abnormal proliferation of synovial cells and MMP-mediated joint structural damage (Paunovic and Harnett, 2013; Baumann et al., 2021).

The principal plant metabolite of *Andrographis paniculata*, andrographolide (AD), suppresses NF-κB and MAPK signaling, inhibits IκBα degradation, diminishes ROS generation, decreases IL-6 and IL-1β levels, and regulates innate and adaptive immunity, suggesting promising therapeutic potential in RA (Zeng et al., 2022; Chen et al., 2025). Curcumin, extracted from the rhizome of *Curcuma longa*, is a natural plant metabolite known for its anti-inflammatory and immunoregulatory properties. Research has shown that curcumin can inhibit TLR4/NF-κB, MAPK, and NLRP3 inflammasome, suppress IKKβ activity, downregulate miR-155, reduce M1 markers such as iNOS and COX-2, and simultaneously activate Nrf2 to inhibit ROS accumulation, induce M2 phenotype polarization, exert anti-inflammatory and antioxidant activities, and effectively alleviate synovial inflammation and cartilage and bone destruction in RA (Mohammadian Haftcheshmeh and Momtazi-Borojeni, 2020; Wang and He, 2022). Nevertheless, existing research suggests that curcumin suffers from poor bioavailability and complex metabolic processing in the body. Oxypeucedanin hydrate (OXH) is a naturally occurring coumarin with notable biological activity. Liu et al. (2024) found that OXH can inhibit the NF-κB/MAPK pathways, reduce the release of proinflammatory cytokines and ROS production, and significantly improve symptoms and bone erosion in CIA models. However, its clinical efficacy still needs to be confirmed through high-quality randomized controlled trials in humans. MAPK signaling exhibits extensive cross-talk with pathways such as NF-κB and JAK-STAT. The multi-target nature of plant metabolite may potentiate anti-inflammatory responses by concurrently regulating several intersecting nodes, but the exact mechanisms of synergy remain to be elucidated.

#### 3.1.4 Toll-like receptors (TLRs) signaling pathway

TLRs, a type of pattern recognition receptor, detect pathogen-associated molecular patterns (PAMPs) and damage-associated molecular patterns (DAMPs) to trigger downstream signaling pathways including NF-κB and MAPK, thereby broadly participating in inflammatory responses and immune modulation (Fitzgerald and Kagan, 2020). In RA patients, TLR expression is significantly upregulated in synovial tissue, particularly TLR2, TLR3, TLR4, and TLR7. These receptors recognize plant metabolites or tissue damage products, activating inflammatory signaling pathways, ultimately leading to excessive synoviocyte proliferation, cytokine release, and bone erosion, and representing one of the initiating events of RA inflammation (Thwaites et al., 2014).

Ginsenoside Rb1, the main active plant metabolite of *Panax ginseng*, exhibits significant anti-inflammatory activity. Experiments have shown that Rb1 reduces the release of inflammatory cytokines and iNOS expression via the TLR4-MyD88-NF-κB/MAPK pathway. In CIA models, mice treated with Rb1 showed markedly decreased TLR4 and MyD88 protein expression in joint tissues, accompanied by alleviated synovial inflammation and improved cartilage damage (Gao et al., 2020; Li W. et al., 2025). Euphorbia factor L2 (EFL2), a diterpenoid plant metabolite derived from *Euphorbia seeds*, has been shown by Tang et al. (2021) in animal studies to specifically target the TLR7 signaling pathway, inhibit IRAK4 autophosphorylation and its interaction with IRF5, block IRF5 nuclear translocation and type I interferon production, suppress TLR7-mediated NF-κB activation, reduce M1 macrophage marker expression, and significantly improve arthritis scores and pathological changes in STA mouse models. These findings offer guidance for the prospective clinical use of this plant metabolites. As upstream targets of inflammation, inhibition of TLRs can block the inflammatory cascade at its source; however, due to functional redundancy among TLR family members, the receptor selectivity of plant metabolite needs to be further clarified.

#### 3.1.5 NLRP3 inflammasome signaling pathway

The NLRP3 inflammasome is a key multiprotein complex widely involved in immune responses and inflammatory regulation. It activates downstream caspase-1 by sensing various danger signals inside and outside cells (such as pathogen-associated molecular patterns, damage-associated molecular patterns, urate crystals, etc.), which in turn promotes the maturation and release of pro-inflammatory cytokines IL-1β and IL-18 (Li et al., 2024), These inflammatory factors further activate effector T cells and M1 macrophages, triggering local and systemic inflammatory responses and exacerbating immune attacks on the joints.

In light of the pivotal role of the NLRP3 inflammasome in the pathology of RA and its status as an inflammatory amplifier, targeting its assembly or activation has become a key strategy in the development of new therapeutic approaches for RA. For example, Baicalin—a flavonoid derived from *Scutellaria baicalensis*—reverses the TLR-2-NF-κB-mediated activation of the NLRP3 inflammasome pathway, inhibits inflammasome assembly, blocks Caspase-1 activation, reduces IL-1β production, and suppresses M1 polarization, thereby exerting antioxidant and anti-inflammatory effects (Hang et al., 2018; Ishfaq et al., 2021). Loganin, a cyclopentanoid monoterpene glycoside derived from *Cornus officinalis*, has been demonstrated in preclinical studies to block NF-κB signaling, attenuate NLRP3 inflammasome activation, inhibit M1 macrophage polarization, and mitigate experimental arthritis through its anti-inflammatory properties (Li et al., 2021; Zhang J. et al., 2021). It is evident that the NLRP3–NF-κB positive feedback loop plays a key role in amplifying inflammation, and the concurrent suppression of these two pathways by plant metabolites may exert synergistic anti-inflammatory benefits. With further validation of clinical efficacy, these plant metabolite may hold promising therapeutic potential in the treatment of RA.

### 3.2 Activation of core anti-inflammatory/antioxidant pathways to promote M2 polarization

#### 3.2.1 Nrf2/HO-1 signaling pathway

Nrf2 (Nuclear Factor Erythroid 2-related Factor 2) is a central transcription factor in the antioxidant response, primarily regulating the expression of downstream antioxidant enzymes and anti-inflammatory proteins by binding to the antioxidant response element (ARE). Examples include heme oxygenase-1 (HO-1), which is a key anti-inflammatory protein that catalyzes the degradation of heme into carbon monoxide (CO), iron ions, and biliverdin, thereby exerting antioxidant and anti-inflammatory effects (Sha et al., 2023). Consequently, the Nrf2/HO-1 signaling pathway is crucial for preserving cellular redox homeostasis, suppressing inflammation, and shielding tissues from oxidative stress-related injury (O'Rourke et al., 2024). In RA, oxidative stress levels are significantly elevated, causing cellular and tissue damage. Activation of Nrf2 can upregulate HO-1, reduce ROS, inhibit the release of inflammatory mediators, and alleviate joint damage (Hung et al., 2024). Additionally, Nrf2/HO-1 pathway activation reduces oxidative stress, inhibits M1 macrophage polarization, and facilitates M2 polarization, with these effects partly achieved via modulation of the TGF-β/SMAD, TLR/NF-κB, and JAK/STAT signaling pathways (Kobayashi et al., 2016).

Asiatic acid (AA), the main active metabolite of the *Centella asiatica*, inhibits the proliferation and colony formation of RA fibroblast-like synoviocytes by activating the Nrf2/HO-1/NF-κB signaling pathway (Zhang L. et al., 2024). Sinomenine (SIN), a metabolite isolated from the botanical drug *Sinomenium acutum*, exhibits anti-inflammatory properties. Guo et al. (2025) and colleagues showed in animal experiments that SIN activates the Nrf2/HO-1 signaling pathway and inhibits NF-κB signaling in mouse chondrocytes, reducing IL-1β-induced inflammatory responses and cartilage degradation, which ameliorates cartilage damage in collagen-induced arthritis (CIA) mouse models, highlighting SIN’s potential new application in RA therapy. Magnoflorin (MAG), the principal plant metabolite from *Cornus officinalis*, has anti-inflammatory, antioxidant, and immunosuppressive activities. Shen et al. (2022) revealed that MAG partly inhibits the PI3K/Akt/NF-κB signaling axis and activates the Keap1-Nrf2/HO-1 pathway, markedly decreasing inflammatory cytokines including iNOS, COX-2, IL-6, and IL-8. This leads to effective suppression of M1 macrophage polarization and promotion of M2 polarization, contributing to its anti-rheumatoid arthritis effects. Likewise, these findings originate from animal-level studies, with the mechanisms remaining unverified in humans. The precise targets of MAG, the pathways of signal cross-regulation, and potential adverse effects warrant further comprehensive research. At the same time, sustained Nrf2 activation could impact normal immune responses, necessitating the development of targeted modulation approaches.

#### 3.2.2 PPARγ signaling pathway

Peroxisome proliferator-activated receptor gamma (PPARγ) is a crucial nuclear receptor involved in the regulation of lipid metabolism and inflammation. As a ligand-dependent transcription factor, it plays an essential role in immune regulation, inflammation suppression, and metabolic balance, with particular importance in the development and treatment of RA (Geng et al., 2024). In the synovial tissue of RA patients, reduced PPARγ expression correlates closely with the proinflammatory status (Li et al., 2023). This phenomenon may be attributed to PPARγ activation inhibiting NF-κB and AP-1 activity, reducing proinflammatory cytokine secretion, and directly promoting M2 gene expressio (Liu S. et al., 2021).

Astragaloside IV (AS-IV), a natural saponin derived from the botanical drug *Astragalus*, can activate the PPARγ pathway to enhance fatty acid oxidation, induce M2 macrophage polarization, and stimulate IL-10 and TGF-β production, thereby promoting macrophage-mediated repair and significantly reducing joint inflammation in RA (Xiong et al., 2025). However, pharmacokinetic and pharmacodynamic studies investigating its efficacy in RA treatment are still lacking. Bavachinin (BVC), a natural plant metabolite isolated from *Cullen corylifolium*, possesses a wide range of pharmacological properties such as antitumor, anti-inflammatory, antioxidant, antimicrobial, antiviral, and immunomodulatory effects. According to Deng et al. (2023), BVC suppresses MH7A cell proliferation, migration, and inflammatory mediator production through the PPARγ/PI3K/AKT signaling pathway, thereby reducing joint injury and inflammation in CIA mice. Unfortunately, no studies to date have reported on its *in vivo* efficacy. Given that PPARγ serves as a central target at the intersection of metabolism and inflammation, its ligands must be developed with consideration for balancing anti-inflammatory and metabolic modulation.

#### 3.2.3 AMPK signaling pathway

AMP-activated protein kinase (AMPK) signaling serves as a key modulator of energy metabolism and plays a broad role in regulating metabolic processes, inflammation, and cell proliferation (Herzig and Shaw, 2018). In RA, AMPK activation can inhibit the abnormal proliferation and migration of RA fibroblast-like synovial cells (RA-FLS), and reduce the release of inflammatory mediators such as TNF-α and IL-6, thereby alleviating joint damage; simultaneously, AMPK can regulate the RANKL-OPG bone protective factor pathway to inhibit bone destruction (Qiu et al., 2021). Moreover, AMPK activation inhibits glycolysis, promotes fatty acid oxidation, and induces M2 polarization by suppressing mTOR (Cui et al., 2023).

Berberine, a prominent anti-inflammatory alkaloid derived from the rhizome of *Coptis chinensis*, acts by activating AMPK, suppressing mTORC1, promoting mitochondrial oxidative phosphorylation, and reducing glycolytic metabolites such as lactate. It induces M2 macrophage polarization and inhibits the NLRP3 inflammasome through AMPK activation, thereby attenuating pyroptosis-associated inflammation and contributing to overall inflammation relief (Cheng et al., 2023). Naringenin, a naturally occurring flavonoid present in numerous plants, exhibits potent anti-inflammatory and immune-regulating properties. According to research by Zhang et al. (2023) and colleagues, Naringenin mitigates joint inflammation in CIA models by activating the AMPK/ULK1 pathway, reducing the release of pro-inflammatory cytokines, and boosting antioxidant defenses. Therefore, the AMPK pathway can integrate metabolic and inflammatory signals, and its activation by plant metabolites may restore immunometabolic homeostasis in RA.

#### 3.2.4 STAT6 signaling pathway

STAT6, an important signaling molecule in the STAT (signal transducer and activator of transcription) family, is mainly activated by cytokines like IL-4 and IL-13. It plays a critical role in immune responses, allergic diseases, and chronic inflammatory diseases (Cai et al., 2019). Evidence indicates that the IL-4/IL-13/STAT6 pathway contributes significantly to RA pathogenesis IL-4 and IL-13 induce STAT6 phosphorylation to promote M2 macrophage polarization, suppress the release of pro-inflammatory mediators, and enhance the expression of markers such as Arg1 and FIZZ1, thereby supporting tissue regeneration and reducing inflammation (Parveen et al., 2025).

The natural flavonoid luteolin possesses potent anti-inflammatory activity; by upregulating p-STAT6 and downregulating p-STAT3, it promotes the transition from M1 to M2 macrophages and effectively alleviates joint inflammation in RA (Gu et al., 2024; Zhu et al., 2024). Suberosin (SBR), extracted from the plant *Plumbago zeylanica L.*, has demonstrated anti-inflammatory and immunomodulatory properties. Liu et al. (2025) and colleagues found in animal experiments that SBR can inhibit JAK1/STAT3 while promoting JAK1/STAT6 phosphorylation, enhance M2 polarization, and alleviate arthritis in CIA mice. In summary, the promise of these plant-derived metabolites necessitates additional clinical evaluation and the development of approaches to address their poor bioavailability. The balanced regulation of STAT6 and STAT3 is critical for maintaining M1/M2 macrophage equilibrium, and the bidirectional modulatory effects of plant metabolites warrant further investigation.

### 3.3 Regulating metabolic reprogramming

Metabolic reprogramming describes alterations in cellular metabolic pathways under certain conditions, influencing cell function, differentiation, and inflammation. In RA, this phenomenon is especially evident, and its mechanisms and therapeutic implications are emerging as key areas of investigation (Jia et al., 2023). Metabolically, M1 macrophages depend mainly on glycolysis. This increased glycolytic activity produces abundant lactate and ATP, leading to the activation of HIF-1α and NF-κB, which in turn promote the production of pro-inflammatory mediators like IL-1β and TNF-α. Conversely, M2 macrophages are more dependent on mitochondrial fatty acid oxidation and oxidative phosphorylation, which fuel the production of anti-inflammatory factors like IL-10 and TGF-β, and mediate repair processes, contributing to inflammation and tissue healing (El Kasmi and Stenmark, 2015; He et al., 2021).

Rtesunate (ARS), a potent antioxidant, exerts anti-arthritic effects by destabilizing HIF-1α, downregulating key glycolytic enzymes, inhibiting glycolysis, thereby suppressing pro-inflammatory cytokine secretion from M1 macrophages; while activating the p62/Nrf2 antioxidant signaling pathway, increasing glutathione production, decreasing reactive oxygen species (ROS) accumulation, and fostering M2 macrophage polarization, thereby mitigating bone erosion in RA (Su et al., 2021). Research by Liu Q. Y. et al. (2021) revealed that Tanshinone IIA, via inactivation of succinate dehydrogenase (SDH), diminishes HIF-1α activation, and maintains Sirt2 activity by suppressing glycolysis, which contributes to the inhibition of NLRP3 inflammasome activation and effectively attenuates inflammation in mouse models, offering a new understanding of its anti-inflammatory mechanism via metabolic and redox modulation. Overall, targeting metabolic reprogramming can simultaneously modulate inflammation and oxidative stress; however, given the complexity of cellular metabolic networks, the specific metabolic targets of plant metabolites need to be clarified.

### 3.4 Modulating epigenetic modifications

Epigenetic modifications refer to mechanisms that regulate gene expression through chemical modifications without altering the DNA sequence, including DNA methylation, RNA methylation, non-coding RNA function, and histone modifications (Gjaltema and Rots, 2020).

Research indicates that epigenetic modifications dynamically reshape chromatin architecture as macrophages polarize from M1 to M2, thereby controlling the activation or repression of genes involved in inflammation and tissue repair (Chen et al., 2020). Plant metabolites can remodel macrophage polarization by targeting key nodes of epigenetic modifications, including DNA methylation/demethylation, histone acetylation/methylation, and non-coding RNAs (Wang W. et al., 2024). Xue et al. (2023) and colleagues demonstrated experimentally that aconitine inhibits NF-κB and NFATc1 activation, reduces osteoclast-specific gene expression, and influences epigenetic reprogramming during macrophage polarization. Currently, research on the epigenetic regulation by plant metabolites is limited. In the future, multi-omics approaches such as ChIP-seq and ATAC-seq could be employed to elucidate the mechanisms by which they remodel the epigenetic landscape.

## 4 Discussion

In this review, we systematically summarized the pivotal functions of M1/M2 macrophages in RA development and progression, as well as highlighted the therapeutic value and significance of targeting macrophage polarization with plant metabolites. At the same time, we have systematically summarized the mechanisms by which plant metabolites treat RA by regulating macrophage polarization, covering major signaling pathways, metabolic reprogramming, and epigenetic modifications. Current studies suggest that these plant metabolites possess multi-target and holistic regulatory advantages; for instance, triptolide and celastrol restore M1/M2 balance by synergistically inhibiting multiple proinflammatory pathways and activating anti-inflammatory pathways, and some researchers have improved the limitations of its very short half-life and adverse GI effects through delivery routes such as nano-delivery and self-assembled hydrogels (Liu et al., 2025; Wang W. et al., 2025). These studies not only reveal how plant metabolites regulate macrophage fate through modulation of key signaling pathways, metabolic reprogramming, and epigenetic modifications, but also provide modern immunological interpretations for the traditional therapeutic principles of “dispelling wind and dampness, promoting blood circulation, and dredging the channels” (Table 1).

**TABLE 1:** Representative plant metabolites regulating M1/M2 macrophage polarization and their mechanisms.

Plant metabolites	Source	Target/pathway	Effect	References
Triptolide	Tripterygium wilfordii Hook.f. [Celastraceae]	NF-κB, MAPK, STAT1	TNF-α↓, IL-6↓, inhibits M1 polarization	Yang et al. (2016), Wang X. et al. (2025)
Glaucocalyxin B, Gla B	Isodon amethystoides (Benth.) H.Hara [Lamiaceae]	p65, p-P65, NF-κB	TNF-α↓, IL-1β↓, IL-6↓, iNOS↓, IL-12↓, inhibiting M1 polarization	Han et al. (2021)
Punicalagin	Punica granatum L. [Lythraceae]	NF-κB	IL-1β↓, IL-18↓, Arg1↑, IL-10↑, inhibiting M1 polarization and promoting M2 polarization	Ge et al. (2022)
Resveratrol	Reynoutria japonica Houtt. [Polygonaceae]	JAK1-STAT1/STAT3	IL-6↓, IL-12↓, inhibiting M1 polarization	Oliveira et al. (2017), Wang P. et al. (2024)
Jolkinolide B, JB	Euphorbia pekinensis Rupr. [Euphorbiaceae]	JAK2/STAT3	Inflammatory cytokines↓, inhibiting M1 polarization	Yan et al. (2024)
Sappanone A, SA	*Tamarindus indica* L. [Fabaceae]	PI3K/AKT/NF-κB, JAK2/STAT3	TNF-α↓, IL-1β↓, IL-6↓, IL-17↓, IL-10↑, inhibiting M1 polarization and promoting M2 polarization	Deng et al. (2024)
Andrographolide,AD	Andrographis paniculata (Burm.f.) Wall. ex Nees [Acanthaceae]	NF-κB, MAPK	ROS↓, IL-6↓, IL-1β↓, inhibiting M1 polarization	Zeng et al. (2022), Chen et al. (2025)
Curcumin	Curcuma longa L. [Zingiberaceae]	TLR4/NF-κB, MAPK, NLRP3, Nrf2	iNOS↓, COX-2↓, ROS↓, IL-6↓, TNF-α↓, inhibiting M1 polarization and inducing M2 polarization	Mohammadian Haftcheshmeh and Momtazi-Borojeni (2020), Wang and He (2022)
Oxypeucedanin hydrate, OXH	Kitagawia praeruptora (Dunn) Pimenov [Apiaceae]	NF-κB/MAPK	iNOS↓, COX-2↓, IL-1β↓, IL-6↓, NF-κB↓, ROS↓, inhibiting M1 polarization	Liu et al. (2024)
Ginsenoside Rb1	Panax ginseng C.A.Mey. [Araliaceae]	TLR4-MyD88-NF-κB/MAPK	iNOS↓, COX-2, ROS↓, NO↓, TNF-α↓, inhibiting M1 polarization and inducing M2 polarization	Gao et al. (2020), Li S. et al. (2025)
Euphorbia factor L2, EFL2	Euphorbia esula subsp. esula [Euphorbiaceae]	TLR7-IRAK4-IKKβ-IRF5/NF-κB	Chemokine mRNA expression↓, pro-inflammatory cytokine production↓, inhibiting M1 polarization	Tang et al. (2021)
Baicalin	Scutellaria baicalensis Georgi [Lamiaceae]	NLRP3, NF-κB	IL-1β↓, IL-10↑, TGF-β↑, inhibiting M1 polarization	Hang et al. (2018), Ishfaq et al. (2021)
Loganin	Cornus officinalis Siebold & Zucc. [Cornaceae]	NF-κB, NLRP3	Inhibiting M1 polarization	Li et al. (2021), Zhang J. et al. (2021)
Asiatic acid,AA	*Centella asiatica* (L.) Urb. [Apiaceae]	Nrf2/HO-1/NF-κB	Inhibiting M1 polarization, inducing M2 polarization	Zhang C. et al. (2024)
Sinomenine, SIN	Sinomenium acutum (Thunb.) Rehder & E.H.Wilson [Menispermaceae]	Nrf2/Ho-1, NF-κB	IL-1β↓, iNOS↓, COX-2↓, NO↓, PGE2↓, TNF-α↓, IL-6↓, inhibiting M1 polarization, promoting M2 polarization	Guo et al. (2025)
Magnoflorine, MAG	Clematis hexapetala Pall. [Ranunculaceae]	PI3K/Akt/NF-κB, Keap1-Nrf2/HO-1	iNOS↓, COX-2↓, IL-6↓, IL-8↓,inhibits M1 polarization, promotes M2 polarization	Shen et al. (2022)
Astragaloside IV	Astragalus mongholicus Bunge [Fabaceae]	PPARγ	IL-10↑, TGF-β↑, inducing M2 polarization	Xiong et al. (2025)
Bavachinin, BVC	Cullen corylifolium (L.) Medik. [Fabaceae]	PPARγ/PI3K/AKT	Inhibition of inflammatory cytokine proliferation↓, inhibiting M1 polarization and promoting M2 polarization	Deng et al. (2023)
Berberine	Coptis chinensis Franch. [Ranunculaceae]	AMPK, NLRP3	Lactic acid↓, promoting M2 polarization	Cheng et al. (2023)
Naringenin	Citrus × aurantium f. aurantium [Rutaceae]	AMPK/ULK1	Reduction in ROS, IL-6, IL-1β, and TNF-α, inhibiting M1 polarization and promoting M2 polarization	Zhang et al. (2023)
Luteolin	Dracocephalum integrifolium Bunge [Lamiaceae]	STAT6, STAT3	IL-6↓, TNF-α↓, IL-10↑, balance M1/M2 polarization	Gu et al. (2024), Zhu et al. (2024)
Suberosin, SBR	Plumbago zeylanica L. [Plumbaginaceae]	JAK1/STAT3, JAK1/STAT6	Inhibit M1 polarization, promote M2 polarization	Liu et al. (2025)
Artesunate, ARS	Artemisia annua L. [Asteraceae]	HIF-1α, Glycolysis, p62/Nrf2	Lactate↓, ROS↓, inhibit M1 polarization, promote M2 polarization	Su et al. (2021)
Tanshinone IIA	Salvia miltiorrhiza Bunge [Lamiaceae]	HIF-1α, Glycolysis, NLRP3	IL-1β↓, IL-6↓, IL-1RA↑, IL-10↑, inhibits M1 polarization, promotes M2 polarization	Liu Q. Y. et al (2021)
Aconitine	Aconitum kusnezoffii Rchb. [Ranunculaceae]	NF-κB, NFATc1, Histone modification	M1/M2 polarization balance	Xue et al. (2023)

Notably, although plant metabolites such as curcumin and resveratrol have been shown *in vitro* to improve RA symptoms by inhibiting macrophage polarization, they are categorized as pan-assay interference compounds (PAINS), which may generate false-positive results *in vitro* through non-specific mechanisms (Bolz et al., 2021). Their specific effects and mechanisms need to be further validated in more physiologically relevant models or *in vivo* studies to exclude assay interference. Moreover, most studies did not provide cell viability data to demonstrate the minimum effective concentration and confirm that these compounds are non-toxic at such concentrations. Therefore, these findings require further validation in primary cells or *in vivo* models. In summary, current studies have several limitations. First, most evidence comes from cellular and animal experiments, with scarce clinical data, particularly lacking pharmacokinetic and human efficacy information. Second, poor oral bioavailability and unstable *in vivo* metabolism of plant metabolites limit clinical translation, necessitating the use of novel delivery systems such as nanoparticles or liposomes to improve druggability. Third, the synergistic or antagonistic relationships among different pathways (e.g., NF-κB and NLRP3, JAK-STAT and AMPK) remain unclear, making precise “pathway network” modulation difficult. Finally, research on the regulation of macrophage metabolism and epigenetics by plant metabolites is still in its infancy, with limited mechanistic depth.

## 5 Conclusions and challenges

Plant metabolites exhibit broad therapeutic potential in RA via multi-target regulation of macrophage polarization, suppressing M1 proinflammatory polarization while enhancing M2 anti-inflammatory and reparative polarization. Their effects involve multiple levels, including signaling pathways, metabolic reprogramming, and epigenetic regulation. Espite abundant preclinical evidence supporting the use of plant metabolites to mitigate RA through multi-target modulation of macrophage polarization, the field faces several challenges: most studies are confined to *in vitro* and animal models, lacking clinical RCT validation for efficacy and safety; pharmacokinetic complexity and low bioavailability constrain clinical translation; research predominantly addresses short-term outcomes, with insufficient data on long-term safety and adverse effects; RA heterogeneity results in variable patient responses, highlighting the need for biomarker-guided precision therapy; moreover, while multi-target mechanisms offer advantages, current studies mainly emphasize transcription factor regulation (e.g., NF-κB, STAT) and downstream inflammatory suppression, lacking comprehensive integration of the “metabolism–epigenetic–immune” network.

To promote clinical translation, future strategies should include: standardizing extraction, quality control, and formulation processes and conducting rigorous clinical trials to verify the efficacy and safety of active plant metabolites; employing novel platforms such as organoids and organ-on-a-chip to better recapitulate human microenvironments and enhance preclinical predictability; performing biomarker-guided precision medicine studies to select optimal patient populations; integrating multi-target datasets via systems biology and computational modeling to decipher pathway crosstalk and network regulation; developing innovative delivery systems to improve bioavailability and targeting; applying advanced technologies such as epigenetics and metabolomics to reveal upstream regulatory mechanisms; and investigating combination therapies of plant metaboliteswith conventional Western medications for synergistic effects. With interdisciplinary collaboration and ongoing research, plant metabolites hold promise for providing novel RA treatment strategies and driving the modernization of traditional Chinese medicine.

## References

[B1] Anders C. B. Lawton T. M. W. Smith H. L. Garret J. Doucette M. M. Ammons M. C. B. (2022). Use of integrated metabolomics, transcriptomics, and signal protein profile to characterize the effector function and associated metabotype of polarized macrophage phenotypes. J. Leukoc. Biol. 111 (3), 667–693. 10.1002/jlb.6a1120-744r 34374126 PMC8825884

[B2] Asmis R. (2016). Monocytes and macrophages: a fresh look at functional and phenotypic diversity. Antioxid. Redox Signal 25 (14), 756–757. 10.1089/ars.2016.6849 27488399 PMC5098125

[B3] Baumann D. Drebant J. Hägele T. Burger L. Serger C. Lauenstein C. (2021). p38 MAPK signaling in M1 macrophages results in selective elimination of M2 macrophages by MEK inhibition. J. Immunother. Cancer 9 (7), e002319. 10.1136/jitc-2020-002319 34285105 PMC8292803

[B4] Bolz S. N. Adasme M. F. Schroeder M. (2021). Toward an understanding of pan-assay interference compounds and promiscuity: a structural perspective on binding modes. J. Chem. Inf. Model 61 (5), 2248–2262. 10.1021/acs.jcim.0c01227 33899463

[B5] Burmester G. R. Pope J. E. (2017). Novel treatment strategies in rheumatoid arthritis. Lancet 389 (10086), 2338–2348. 10.1016/s0140-6736(17)31491-5 28612748

[B6] Cai W. Dai X. Chen J. Zhao J. Xu M. Zhang L. (2019). STAT6/Arg1 promotes microglia/macrophage efferocytosis and inflammation resolution in stroke mice. JCI Insight 4 (20), e131355. 10.1172/jci.insight.131355 31619589 PMC6824303

[B7] Chambers M. Rees A. Cronin J. G. Nair M. Jones N. Thornton C. A. (2020). Macrophage plasticity in reproduction and environmental influences on their function. Front. Immunol. 11, 607328. 10.3389/fimmu.2020.607328 33519817 PMC7840613

[B8] Chen T. Cao Q. Wang Y. Harris D. C. H. (2019). M2 macrophages in kidney disease: biology, therapies, and perspectives. Kidney Int. 95 (4), 760–773. 10.1016/j.kint.2018.10.041 30827512

[B9] Chen S. Yang J. Wei Y. Wei X. (2020). Epigenetic regulation of macrophages: from homeostasis maintenance to host defense. Cell Mol. Immunol. 17 (1), 36–49. 10.1038/s41423-019-0315-0 31664225 PMC6952359

[B10] Chen S. Zeng J. Li R. Zhang Y. Tao Y. Hou Y. (2024). Traditional Chinese medicine in regulating macrophage polarization in immune response of inflammatory diseases. J. Ethnopharmacol. 325, 117838. 10.1016/j.jep.2024.117838 38310986

[B11] Chen Y. He P. Tao S. Zhong J. Jiang K. Hsu Y. (2025). Injectable sustainable andrographolide-releasing hydrogel for long-lasting alleviation of osteoarthritis and regulation of chondrocyte autophagy via PRKCA/EGFR. Mater Today Bio 31, 101610. 10.1016/j.mtbio.2025.101610 40104642 PMC11919379

[B12] Cheng J. W. Yu Y. Zong S. Y. Cai W. W. Wang Y. Song Y. N. (2023). Berberine ameliorates collagen-induced arthritis in mice by restoring macrophage polarization via AMPK/mTORC1 pathway switching glycolytic reprogramming. Int. Immunopharmacol. 124 (Pt B), 111024. 10.1016/j.intimp.2023.111024 37827054

[B13] Cui Y. Chen J. Zhang Z. Shi H. Sun W. Yi Q. (2023). The role of AMPK in macrophage metabolism, function and polarisation. J. Transl. Med. 21 (1), 892. 10.1186/s12967-023-04772-6 38066566 PMC10709986

[B14] Cutolo M. Campitiello R. Gotelli E. Soldano S. (2022). The role of M1/M2 macrophage polarization in rheumatoid arthritis synovitis. Front. Immunol. 13, 867260. 10.3389/fimmu.2022.867260 35663975 PMC9161083

[B15] Cutolo M. Soldano S. Smith V. Gotelli E. Hysa E. (2025). Dynamic macrophage phenotypes in autoimmune and inflammatory rheumatic diseases. Nat. Rev. Rheumatol. 21 (9), 546–565. 10.1038/s41584-025-01279-w 40721670

[B16] Deng H. Jiang J. Shu J. Huang M. Zhang Q. L. Wu L. J. (2023). Bavachinin ameliorates rheumatoid arthritis inflammation via PPARG/PI3K/AKT signaling pathway. Inflammation 46 (5), 1981–1996. 10.1007/s10753-023-01855-w 37358659

[B17] Deng C. Sun S. Zhang H. Liu S. Xu X. Hu Y. (2024). Sappanone A attenuates rheumatoid arthritis via inhibiting PI3K/AKT/NF-κB and JAK2/STAT3 signaling pathways *in vivo* and *in vitro* . Int. Immunopharmacol. 143 (Pt 3), 113560. 10.1016/j.intimp.2024.113560 39520962

[B18] Deng Y. Li B. Zheng H. Liang L. Yang Y. Liu S. (2025). Multifunctional Prussian blue nanoparticles loading with Xuetongsu for efficient rheumatoid arthritis therapy through targeting inflammatory macrophages and osteoclasts. Asian J. Pharm. Sci. 20 (3), 101037. 10.1016/j.ajps.2025.101037 40503057 PMC12152339

[B19] Dey A. Allen J. Hankey-Giblin P. A. (2014). Ontogeny and polarization of macrophages in inflammation: blood monocytes versus tissue macrophages. Front. Immunol. 5, 683. 10.3389/fimmu.2014.00683 25657646 PMC4303141

[B20] El Kasmi K. C. Stenmark K. R. (2015). Contribution of metabolic reprogramming to macrophage plasticity and function. Semin. Immunol. 27 (4), 267–275. 10.1016/j.smim.2015.09.001 26454572 PMC4677817

[B21] Fitzgerald K. A. Kagan J. C. (2020). Toll-like receptors and the control of immunity. Cell 180 (6), 1044–1066. 10.1016/j.cell.2020.02.041 32164908 PMC9358771

[B22] Fleischmann R. M. van der Heijde D. Strand V. Atsumi T. McInnes I. B. Takeuchi T. (2023). Anti-GM-CSF otilimab versus tofacitinib or placebo in patients with active rheumatoid arthritis and an inadequate response to conventional or biologic DMARDs: two phase 3 randomised trials (contRAst 1 and contRAst 2). Ann. Rheum. Dis. 82 (12), 1516–1526. 10.1136/ard-2023-224482 37699654 PMC10646845

[B23] Fortelny N. Farlik M. Fife V. Gorki A. D. Lassnig C. Maurer B. (2024). JAK-STAT signaling maintains homeostasis in T cells and macrophages. Nat. Immunol. 25 (5), 847–859. 10.1038/s41590-024-01804-1 38658806 PMC11065702

[B24] Gao H. Kang N. Hu C. Zhang Z. Xu Q. Liu Y. (2020). Ginsenoside Rb1 exerts anti-inflammatory effects *in vitro* and *in vivo* by modulating toll-like receptor 4 dimerization and NF-kB/MAPKs signaling pathways. Phytomedicine 69, 153197. 10.1016/j.phymed.2020.153197 32146298

[B25] Ge G. Bai J. Wang Q. Liang X. Tao H. Chen H. (2022). Punicalagin ameliorates collagen-induced arthritis by downregulating M1 macrophage and pyroptosis via NF-κB signaling pathway. Sci. China Life Sci. 65 (3), 588–603. 10.1007/s11427-020-1939-1 34125371

[B26] Geng Q. Xu J. Cao X. Wang Z. Jiao Y. Diao W. (2024). PPARG-mediated autophagy activation alleviates inflammation in rheumatoid arthritis. J. Autoimmun. 146, 103214. 10.1016/j.jaut.2024.103214 38648706

[B27] Gjaltema R. A. F. Rots M. G. (2020). Advances of epigenetic editing. Curr. Opin. Chem. Biol. 57, 75–81. 10.1016/j.cbpa.2020.04.020 32619853

[B28] Gravallese E. M. Firestein G. S. Koscal N. Ling E. Longo D. L. Messenger L. A. (2024). What is rheumatoid arthritis? N. Engl. J. Med. 390 (13), e32. 10.1056/NEJMp2310178 38598569

[B29] Gu J. Zhang P. Li H. Wang Y. Huang Y. Fan L. (2024). Cerium-Luteolin nanocomplexes in managing inflammation-related diseases by antioxidant and immunoregulation. ACS Nano 18 (8), 6229–6242. 10.1021/acsnano.3c09528 38345570

[B30] Guo Q. Jin Y. Chen X. Ye X. Shen X. Lin M. (2024). NF-κB in biology and targeted therapy: new insights and translational implications. Signal Transduct. Target Ther. 9 (1), 53. 10.1038/s41392-024-01757-9 38433280 PMC10910037

[B31] Guo W. Y. Wu Q. M. Zeng H. F. Chen Y. L. Xu J. Yu Z. Y. (2025). A sinomenine derivative alleviates bone destruction in collagen-induced arthritis mice by suppressing mitochondrial dysfunction and oxidative stress via the NRF2/HO-1/NQO1 signaling pathway. Pharmacol. Res. 215, 107686. 10.1016/j.phrs.2025.107686 40088961

[B32] Han C. Yang Y. Sheng Y. Wang J. Zhou X. Li W. (2021). Glaucocalyxin B inhibits cartilage inflammatory injury in rheumatoid arthritis by regulating M1 polarization of synovial macrophages through NF-κB pathway. Aging (Albany NY) 13 (18), 22544–22555. 10.18632/aging.203567 34580236 PMC8507279

[B33] Hang Y. Qin X. Ren T. Cao J. (2018). Baicalin reduces blood lipids and inflammation in patients with coronary artery disease and rheumatoid arthritis: a randomized, double-blind, placebo-controlled trial. Lipids Health Dis. 17 (1), 146. 10.1186/s12944-018-0797-2 29935544 PMC6015450

[B34] Hanlon M. M. Smith C. M. Canavan M. Neto N. G. B. Song Q. Lewis M. J. (2024). Loss of synovial tissue macrophage homeostasis precedes rheumatoid arthritis clinical onset. Sci. Adv. 10 (39), eadj1252. 10.1126/sciadv.adj1252 39321281 PMC11423874

[B35] He L. Jhong J. H. Chen Q. Huang K. Y. Strittmatter K. Kreuzer J. (2021). Global characterization of macrophage polarization mechanisms and identification of M2-type polarization inhibitors. Cell Rep. 37 (5), 109955. 10.1016/j.celrep.2021.109955 34731634 PMC8783961

[B36] Herzig S. Shaw R. J. (2018). AMPK: guardian of metabolism and mitochondrial homeostasis. Nat. Rev. Mol. Cell Biol. 19 (2), 121–135. 10.1038/nrm.2017.95 28974774 PMC5780224

[B37] Hu L. Liu R. Zhang L. (2022). Advance in bone destruction participated by JAK/STAT in rheumatoid arthritis and therapeutic effect of JAK/STAT inhibitors. Int. Immunopharmacol. 111, 109095. 10.1016/j.intimp.2022.109095 35926270

[B38] Huang L. Huang X. H. Yang X. Hu J. Q. Zhu Y. Z. Yan P. Y. (2024). Novel nano-drug delivery system for natural products and their application. Pharmacol. Res. 201, 107100. 10.1016/j.phrs.2024.107100 38341055

[B39] Hung S. Y. Chen J. L. Tu Y. K. Tsai H. Y. Lu P. H. Jou I. M. (2024). Isoliquiritigenin inhibits apoptosis and ameliorates oxidative stress in rheumatoid arthritis chondrocytes through the Nrf2/HO-1-mediated pathway. Biomed. Pharmacother. 170, 116006. 10.1016/j.biopha.2023.116006 38091640

[B40] Ishfaq M. Wu Z. Wang J. Li R. Chen C. Li J. (2021). Baicalin alleviates mycoplasma gallisepticum-induced oxidative stress and inflammation via modulating NLRP3 inflammasome-autophagy pathway. Int. Immunopharmacol. 101 (Pt B), 108250. 10.1016/j.intimp.2021.108250 34656906

[B41] Jia N. Gao Y. Li M. Liang Y. Li Y. Lin Y. (2023). Metabolic reprogramming of proinflammatory macrophages by target delivered roburic acid effectively ameliorates rheumatoid arthritis symptoms. Signal Transduct. Target Ther. 8 (1), 280. 10.1038/s41392-023-01499-0 37500654 PMC10374631

[B42] Kang S. Kumanogoh A. (2020). The spectrum of macrophage activation by immunometabolism. Int. Immunol. 32 (7), 467–473. 10.1093/intimm/dxaa017 32179900

[B43] Kobayashi E. H. Suzuki T. Funayama R. Nagashima T. Hayashi M. Sekine H. (2016). Nrf2 suppresses macrophage inflammatory response by blocking proinflammatory cytokine transcription. Nat. Commun. 7, 11624. 10.1038/ncomms11624 27211851 PMC4879264

[B44] Li Q. Hu S. Huang L. Zhang J. Cao G. (2021). Evaluating the therapeutic mechanisms of selected active compounds in Cornus officinalis and Paeonia Lactiflora in rheumatoid arthritis via network pharmacology analysis. Front. Pharmacol. 12, 648037. 10.3389/fphar.2021.648037 33967784 PMC8097135

[B45] Li X. F. Yin S. Q. Li H. Yang Y. L. Chen X. Song B. (2023). PPAR-γ alleviates the inflammatory response in TNF-α-induced fibroblast-like synoviocytes by binding to p53 in rheumatoid arthritis. Acta Pharmacol. Sin. 44 (2), 454–464. 10.1038/s41401-022-00957-9 35918412 PMC9889328

[B46] Li W. He H. Du M. Gao M. Sun Q. Wang Y. (2024). Quercetin as a promising intervention for rat osteoarthritis by decreasing M1-polarized macrophages via blocking the TRPV1-mediated P2X7/NLRP3 signaling pathway. Phytother. Res. 38 (4), 1990–2006. 10.1002/ptr.8158 38372204

[B47] Li Q. Zhao X. Wang A. Hang T. Zhao J. Zhang S. (2025). From molecular mechanism to plant intervention: the bidirectional regulation of inflammation and oxidative stress in bone aging. Front. Endocrinol. (Lausanne) 16, 1634580. 10.3389/fendo.2025.1634580 40704144 PMC12283317

[B48] Li W. Wang Y. Mu W. Guan Y. Yang Y. Tang Y. (2025). Ginsenoside RB1 influences Macrophage-DPSC interactions in inflammatory conditions. Int. Dent. J. 75 (2), 1194–1202. 10.1016/j.identj.2024.07.1213 39191604 PMC11976592

[B49] Lin W. Shen P. Huang Y. Han L. Ba X. Huang Y. (2023). Wutou decoction attenuates the synovial inflammation of collagen-induced arthritis rats via regulating macrophage M1/M2 type polarization. J. Ethnopharmacol. 301, 115802. 10.1016/j.jep.2022.115802 36209953

[B50] Liu Q. Y. Zhuang Y. Song X. R. Niu Q. Sun Q. S. Li X. N. (2021). Tanshinone IIA prevents LPS-induced inflammatory responses in mice via inactivation of succinate dehydrogenase in macrophages. Acta Pharmacol. Sin. 42 (6), 987–997. 10.1038/s41401-020-00535-x 33028985 PMC8149828

[B51] Liu S. S. Zhang H. Li Y. Zhang Y. Bian Y. Zeng Y. (2021). S100A4 enhances protumor macrophage polarization by control of PPAR-γ-dependent induction of fatty acid oxidation. J. Immunother. Cancer 9 (6), e002548. 10.1136/jitc-2021-002548 34145030 PMC8215236

[B52] Liu M. Huo X. Li C. Hu Y. Lei H. Wang D. (2024). Oxypeucedanin hydrate alleviates rheumatoid arthritis by inhibiting the TLR4-MD2/NF-κB/MAPK signaling axis. Acta Biochim. Biophys. Sin. (Shanghai) 56 (12), 1789–1801. 10.3724/abbs.2024076 38734936 PMC11659794

[B53] Liu H. Li Q. Chen Y. Dong M. Liu H. Zhang J. (2025). Suberosin attenuates rheumatoid arthritis by repolarizing macrophages and inhibiting synovitis via the JAK/STAT signaling pathway. Arthritis Res. Ther. 27 (1), 12. 10.1186/s13075-025-03481-3 39838477 PMC11748358

[B54] Locati M. Curtale G. Mantovani A. (2020). Diversity, mechanisms, and significance of macrophage plasticity. Annu. Rev. Pathol. 15, 123–147. 10.1146/annurev-pathmechdis-012418-012718 31530089 PMC7176483

[B55] Ma W. T. Gao F. Gu K. Chen D. K. (2019). The role of monocytes and macrophages in autoimmune diseases: a comprehensive review. Front. Immunol. 10, 1140. 10.3389/fimmu.2019.01140 31178867 PMC6543461

[B56] Marques O. Horvat N. K. Zechner L. Colucci S. Sparla R. Zimmermann S. (2025). Inflammation-driven NF-κB signaling represses ferroportin transcription in macrophages via HDAC1 and HDAC3. Blood 145 (8), 866–880. 10.1182/blood.2023023417 39656097

[B57] McInnes I. B. Schett G. (2011). The pathogenesis of rheumatoid arthritis. N. Engl. J. Med. 365 (23), 2205–2219. 10.1056/NEJMra1004965 22150039

[B58] Mohammadian Haftcheshmeh S. Momtazi-Borojeni A. A. (2020). Immunomodulatory therapeutic effects of curcumin in rheumatoid arthritis. Autoimmun. Rev. 19 (8), 102593. 10.1016/j.autrev.2020.102593 32540449

[B59] O'Rourke S. A. Shanley L. C. Dunne A. (2024). The Nrf2-HO-1 system and inflammaging. Front. Immunol. 15, 1457010. 10.3389/fimmu.2024.1457010 39380993 PMC11458407

[B60] Oliveira A. L. B. Monteiro V. V. S. Navegantes-Lima K. C. Reis J. F. Gomes R. S. Rodrigues D. V. S. (2017). Resveratrol role in autoimmune Disease-A mini-review. Nutrients 9 (12), 1306. 10.3390/nu9121306 29194364 PMC5748756

[B61] Orecchioni M. Ghosheh Y. Pramod A. B. Ley K. (2019). Macrophage polarization: different gene signatures in M1(LPS+) vs. classically and M2(LPS-) vs. alternatively activated macrophages. Front. Immunol. 10, 1084. 10.3389/fimmu.2019.01084 31178859 PMC6543837

[B62] Parveen S. Fatma M. Mir S. S. Dermime S. Uddin S. (2025). JAK-STAT signaling in autoimmunity and cancer. Immunotargets Ther. 14, 523–554. 10.2147/itt.S485670 40376194 PMC12080488

[B63] Paunovic V. Harnett M. M. (2013). Mitogen-activated protein kinases as therapeutic targets for rheumatoid arthritis. Drugs 73 (2), 101–115. 10.1007/s40265-013-0014-6 23371304

[B64] Peng M. Qiang L. Xu Y. Li C. Li T. Wang J. (2019). IL-35 ameliorates collagen-induced arthritis by promoting TNF-α-induced apoptosis of synovial fibroblasts and stimulating M2 macrophages polarization. Febs J. 286 (10), 1972–1985. 10.1111/febs.14801 30834683

[B65] Qiu J. Wu B. Goodman S. B. Berry G. J. Goronzy J. J. Weyand C. M. (2021). Metabolic control of autoimmunity and tissue inflammation in rheumatoid arthritis. Front. Immunol. 12, 652771. 10.3389/fimmu.2021.652771 33868292 PMC8050350

[B66] Samra S. Bergerson J. R. E. Freeman A. F. Turvey S. E. (2025). JAK-STAT signaling pathway, immunodeficiency, inflammation, immune dysregulation, and inborn errors of immunity. J. Allergy Clin. Immunol. 155 (2), 357–367. 10.1016/j.jaci.2024.09.020 39369964

[B67] Satoh T. Akira S. (2013). Physiological role and differentiation mechanism of various M2 macrophages. Japanese J. Allergol. 62 (12), 1575–1582. 10.15036/arerugi.62.1575 24608646

[B68] Sha W. Zhao B. Wei H. Yang Y. Yin H. Gao J. (2023). Astragalus polysaccharide ameliorates vascular endothelial dysfunction by stimulating macrophage M2 polarization via potentiating Nrf2/HO-1 signaling pathway. Phytomedicine 112, 154667. 10.1016/j.phymed.2023.154667 36842218

[B69] Shapouri-Moghaddam A. Mohammadian S. Vazini H. Taghadosi M. Esmaeili S. A. Mardani F. (2018). Macrophage plasticity, polarization, and function in health and disease. J. Cell Physiol. 233 (9), 6425–6440. 10.1002/jcp.26429 29319160 PMC13482560

[B70] Shen Y. Fan X. Qu Y. Tang M. Huang Y. Peng Y. (2022). Magnoflorine attenuates inflammatory responses in RA by regulating the PI3K/Akt/NF-κB and Keap1-Nrf2/HO-1 signalling pathways *in vivo* and *in vitro* . Phytomedicine 104, 154339. 10.1016/j.phymed.2022.154339 35870375

[B71] Sheu K. M. Hoffmann A. (2022). Functional hallmarks of healthy macrophage responses: their regulatory basis and disease relevance. Annu. Rev. Immunol. 40, 295–321. 10.1146/annurev-immunol-101320-031555 35471841 PMC10074967

[B72] Simon L. S. Taylor P. C. Choy E. H. Sebba A. Quebe A. Knopp K. L. (2021). The Jak/STAT pathway: a focus on pain in rheumatoid arthritis. Semin. Arthritis Rheum. 51 (1), 278–284. 10.1016/j.semarthrit.2020.10.008 33412435

[B73] Smigiel K. S. Parks W. C. (2018). Macrophages, wound healing, and fibrosis: recent insights. Curr. Rheumatol. Rep. 20 (4), 17. 10.1007/s11926-018-0725-5 29550962

[B74] Smith M. H. Berman J. R. (2022). What is rheumatoid arthritis? Jama 327 (12), 1194. 10.1001/jama.2022.0786 35315883

[B75] Smolen J. S. Aletaha D. McInnes I. B. (2016). Rheumatoid arthritis. Lancet 388 (10055), 2023–2038. 10.1016/s0140-6736(16)30173-8 27156434

[B76] Smolen J. S. Landewé R. B. M. Bergstra S. A. Kerschbaumer A. Sepriano A. Aletaha D. (2023). EULAR recommendations for the management of rheumatoid arthritis with synthetic and biological disease-modifying antirheumatic drugs: 2022 update. Ann. Rheum. Dis. 82 (1), 3–18. 10.1136/ard-2022-223356 36357155

[B77] Su X. Guo W. Yuan B. Wang D. Liu L. Wu X. (2021). Artesunate attenuates bone erosion in rheumatoid arthritis by suppressing reactive oxygen species via activating p62/Nrf2 signaling. Biomed. Pharmacother. 137, 111382. 10.1016/j.biopha.2021.111382 33761603

[B78] Tang J. Cheng X. Yi S. Zhang Y. Tang Z. Zhong Y. (2021). Euphorbia factor L2 ameliorates the progression of K/BxN serum-induced arthritis by blocking TLR7 mediated IRAK4/IKKβ/IRF5 and NF-kB signaling pathways. Front. Pharmacol. 12, 773592. 10.3389/fphar.2021.773592 34950033 PMC8691750

[B79] Tao J. Yang P. Gao M. Zhang F. Wu Y. Jiang Y. (2023). Reversing inflammatory microenvironment by a single intra-articular injection of multi-stimulus responsive lipogel to relieve rheumatoid arthritis and promote joint repair. Mater Today Bio 20, 100622. 10.1016/j.mtbio.2023.100622 37056918 PMC10085779

[B80] Tardito S. Martinelli G. Soldano S. Paolino S. Pacini G. Patane M. (2019). Macrophage M1/M2 polarization and rheumatoid arthritis: a systematic review. Autoimmun. Rev. 18 (11), 102397. 10.1016/j.autrev.2019.102397 31520798

[B81] Thwaites R. Chamberlain G. Sacre S. (2014). Emerging role of endosomal toll-like receptors in rheumatoid arthritis. Front. Immunol. 5, 1. 10.3389/fimmu.2014.00001 24474949 PMC3893714

[B82] Wang L. He C. (2022). Nrf2-mediated anti-inflammatory polarization of macrophages as therapeutic targets for osteoarthritis. Front. Immunol. 13, 967193. 10.3389/fimmu.2022.967193 36032081 PMC9411667

[B83] Wang C. Ma C. Gong L. Guo Y. Fu K. Zhang Y. (2021). Macrophage polarization and its role in liver disease. Front. Immunol. 12, 803037. 10.3389/fimmu.2021.803037 34970275 PMC8712501

[B84] Wang P. Li Z. Song Y. Zhang B. Fan C. (2024). Resveratrol-driven macrophage polarization: unveiling mechanisms and therapeutic potential. Front. Pharmacol. 15, 1516609. 10.3389/fphar.2024.1516609 39872049 PMC11770351

[B85] Wang W. W. Li H. Shi Y. Zhou J. Khan G. J. Zhu J. (2024). Targeted intervention of natural medicinal active ingredients and traditional Chinese medicine on epigenetic modification: possible strategies for prevention and treatment of atherosclerosis. Phytomedicine 122, 155139. 10.1016/j.phymed.2023.155139 37863003

[B86] Wang W. W. Wang Z. Ling A. Zhang C. Lv M. Huang L. (2025). Research progress in treatment of rheumatoid arthritis with sinomenine and related formulations based on different administration routes. Front. Pharmacol. 16, 1613679. 10.3389/fphar.2025.1613679 40843369 PMC12364828

[B87] Wang X. Ni T. Miao J. Huang X. Feng Z. (2025). The role and mechanism of triptolide, a potential new DMARD, in the treatment of rheumatoid arthritis. Ageing Res. Rev. 104, 102643. 10.1016/j.arr.2024.102643 39722411

[B88] Willenborg S. Lucas T. van Loo G. Knipper J. A. Krieg T. Haase I. (2012). CCR2 recruits an inflammatory macrophage subpopulation critical for angiogenesis in tissue repair. Blood 120 (3), 613–625. 10.1182/blood-2012-01-403386 22577176

[B89] Xiong B. B. Zhuo Y. M. Wang H. Zheng Q. L. Tang F. Huang Q. (2025). Macrophage polarization in disease therapy: insights from astragaloside IV and cycloastragenol. Front. Pharmacol. 16, 1598022. 10.3389/fphar.2025.1598022 40487409 PMC12141326

[B90] Xue C. Luo H. Wang L. Deng Q. Kui W. Da W. (2023). Aconine attenuates osteoclast-mediated bone resorption and ferroptosis to improve osteoporosis via inhibiting NF-κB signaling. Front. Endocrinol. (Lausanne) 14, 1234563. 10.3389/fendo.2023.1234563 38034017 PMC10682992

[B91] Yan Y. Zhang L. B. Ma R. Wang M. N. He J. Wang P. P. (2024). Jolkinolide B ameliorates rheumatoid arthritis by regulating the JAK2/STAT3 signaling pathway. Phytomedicine 124, 155311. 10.1016/j.phymed.2023.155311 38199156

[B92] Yang Y. Ye Y. Qiu Q. Xiao Y. Huang M. Shi M. (2016). Triptolide inhibits the migration and invasion of rheumatoid fibroblast-like synoviocytes by blocking the activation of the JNK MAPK pathway. Int. Immunopharmacol. 41, 8–16. 10.1016/j.intimp.2016.10.005 27816728

[B93] Yang X. Li J. Xu C. Zhang G. Che X. Yang J. (2024). Potential mechanisms of rheumatoid arthritis therapy: focus on macrophage polarization. Int. Immunopharmacol. 142 (Pt A), 113058. 10.1016/j.intimp.2024.113058 39236455

[B94] Yue Y. Yang X. Feng K. Wang L. Hou J. Mei B. (2017). M2b macrophages reduce early reperfusion injury after myocardial ischemia in mice: a predominant role of inhibiting apoptosis via A20. Int. J. Cardiol. 245, 228–235. 10.1016/j.ijcard.2017.07.085 28764858

[B95] Zeng B. Wei A. Zhou Q. Yuan M. Lei K. Liu Y. (2022). Andrographolide: a review of its pharmacology, pharmacokinetics, toxicity and clinical trials and pharmaceutical researches. Phytother. Res. 36 (1), 336–364. 10.1002/ptr.7324 34818697

[B96] Zhang Q. Lenardo M. J. Baltimore D. (2017). 30 years of NF-κB: a blossoming of relevance to human pathobiology. Cell 168 (1-2), 37–57. 10.1016/j.cell.2016.12.012 28086098 PMC5268070

[B97] Zhang J. Wang C. Wang H. Li X. Xu J. Yu K. (2021). Loganin alleviates sepsis-induced acute lung injury by regulating macrophage polarization and inhibiting NLRP3 inflammasome activation. Int. Immunopharmacol. 95, 107529. 10.1016/j.intimp.2021.107529 33744777

[B98] Zhang Y. Mao X. Li W. Chen W. Wang X. Ma Z. (2021). Tripterygium wilfordii: an inspiring resource for rheumatoid arthritis treatment. Med. Res. Rev. 41 (3), 1337–1374. 10.1002/med.21762 33296090

[B99] Zhang W. Zhang Y. Zhang J. Deng C. Zhang C. (2023). Naringenin ameliorates collagen-induced arthritis through activating AMPK-mediated autophagy in macrophages. Immun. Inflamm. Dis. 11 (10), e983. 10.1002/iid3.983 37904715 PMC10588338

[B100] Zhang C. Weng Y. Wang H. Zhan S. Li C. Zheng D. (2024). A synergistic effect of triptolide and curcumin on rheumatoid arthritis by improving cell proliferation and inducing cell apoptosis via inhibition of the IL-17/NF-κB signaling pathway. Int. Immunopharmacol. 142 (Pt A), 112953. 10.1016/j.intimp.2024.112953 39226828

[B101] Zhang L. L. Liu Z. N. Han X. Y. Liu X. Li Y. (2024). Asiatic acid inhibits rheumatoid arthritis fibroblast-like synoviocyte growth through the Nrf2/HO-1/NF-κB signaling pathway. Chem. Biol. Drug Des. 103 (3), e14454. 10.1111/cbdd.14454 38477392

[B102] Zhao W. Ma L. Deng D. Zhang T. Han L. Xu F. (2023). M2 macrophage polarization: a potential target in pain relief. Front. Immunol. 14, 1243149. 10.3389/fimmu.2023.1243149 37705982 PMC10497114

[B103] Zheng Y. Wei K. Jiang P. Zhao J. Shan Y. Shi Y. (2024). Macrophage polarization in rheumatoid arthritis: signaling pathways, metabolic reprogramming, and crosstalk with synovial fibroblasts. Front. Immunol. 15, 1394108. 10.3389/fimmu.2024.1394108 38799455 PMC11116671

[B104] Zhou R. Xue S. Cheng Y. Chen Y. Wang Y. Xing J. (2024). Macrophage membrane-camouflaged biomimetic nanoparticles for rheumatoid arthritis treatment via modulating macrophage polarization. J. Nanobiotechnology 22 (1), 578. 10.1186/s12951-024-02822-9 39300463 PMC11414146

[B105] Zhou G. L. Su S. L. Yu L. Shang E. X. Hua Y. Q. Yu H. (2025). Exploring the liver toxicity mechanism of Tripterygium wilfordii extract based on metabolomics, network pharmacological analysis and experimental validation. J. Ethnopharmacol. 337 (Pt 2), 118888. 10.1016/j.jep.2024.118888 39368758

[B106] Zhu M. Sun Y. Su Y. Guan W. Wang Y. Han J. (2024). Luteolin: a promising multifunctional natural flavonoid for human diseases. Phytother. Res. 38 (7), 3417–3443. 10.1002/ptr.8217 38666435

[B107] Zhu H. Wang Z. Cui J. Ge Y. Yan M. Wu X. (2025). PEGylated retinoate prodrug self-assembled nanomicelles loaded with triptolide for targeting treatment of rheumatoid arthritis and side effect attenuation. Colloids Surf. B Biointerfaces 251, 114618. 10.1016/j.colsurfb.2025.114618 40090173

